# Education Into Policy: Embedding Health Informatics to Prepare Future Nurses—New Zealand Case Study

**DOI:** 10.2196/16186

**Published:** 2020-01-09

**Authors:** Michelle Honey, Emma Collins, Sally Britnell

**Affiliations:** 1 School of Nursing University of Auckland Auckland New Zealand; 2 School of Nursing Otago Polytechnic Dunedin New Zealand; 3 Nursing Department Auckland University of Technology Auckland New Zealand

**Keywords:** informatics, nursing, education

## Abstract

**Background:**

Preparing emerging health professionals for practicing in an ever-changing health care environment along with continually evolving technology is an international concern. This is particularly pertinent for nursing because nurses make up the largest part of the health workforce.

**Objective:**

This study aimed to explore how health informatics can be included in undergraduate health professional education.

**Methods:**

A case study approach was used to consider health informatics within undergraduate nursing education in New Zealand. This has led to the development of nursing informatics guidelines for nurses entering practice.

**Results:**

The process used to develop nursing informatics guidelines for entry to practice in New Zealand is described. The final guidelines are based on the literature and are refined using an advisory group and an iterative process.

**Conclusions:**

Although this study describes the development of nursing informatics guidelines for nurses entering practice, the challenge is to move these guidelines from educational rhetoric to policy. It is only by ensuring that health informatics is embedded in the undergraduate education of all health professionals can we be assured that future health professionals are prepared to work effectively, efficiently, and safely with information and communication technologies as part of their practice.

## Introduction

### Focus of Study

Health professionals work with people of all ages and stages of life; across primary, secondary, and tertiary care; and in a variety of settings, from hospitals to the community [1]. Modern health care often includes the use of technology, which is frequently mirrored in health strategy [2]. For example, the New Zealand Health Strategy calls for health care professionals to work “smarter” using technology to enhance the health and well-being of New Zealanders [3]. There is a general consensus that people will continue to be at the center of any successful digital health initiative [4,5]. This is particularly pertinent for nursing because nurses make up the largest component of the health workforce [6]. Focusing and investing in nursing is thought to improve health and gender equality and support the economic growth of a country [7]. Williamson and Muckle [8] considered technology an integral part of current nursing practice and therefore suggested the use of technology being integrated into the nursing curriculum for students. Although a systematic review suggests that learning mediated by technology may not be better than traditional approaches to teaching and learning, exposure to technology may help develop information and communication technologies (ICT) skills that can be transferred to the clinical setting [9].

The focus of this case study was nurses working in New Zealand. New Zealand is a small country situated deep in the South Pacific with a population of less than 5 million [10]. There are 52,700 practicing nurses or 1106.9 practicing nurses per 100,000 New Zealanders [1]. Nurses are recognized as the largest regulated health workforce in New Zealand, and because of having a generalist scope, diversity, flexibility, and demographic spread, they are the health professionals who are best able to provide a rapid response to emerging health needs [11]. Although historically New Zealand was an early leader in considering how to prepare nurses to work with technology, nursing informatics has not been consistently addressed in nursing curricula across the country’s 17 schools of nursing, meaning that New Zealand nurses may not be well prepared in this area [12-15]. Therefore, a project was established to address this gap.

### Background

There are many terms concerning the use of ICT in health care, but in this instance, the term health informatics, or when specifically for nursing—nursing informatics, has been selected. Health informatics is defined as the discipline focused on the acquisition, storage, and use of information in a specific setting or domain, in this case health care [16]. Within nursing, the term nursing informatics is preferred, and this is defined as a “science and practice [which] integrates nursing, its information and knowledge, and their management, with ICT to promote the health of people, families and communities worldwide” [17]. Or more simply, we are talking about the use of computers and ICT to support health care.

Including health informatics within health professional educational preparation was noted in the literature from the early 1970s; however, there seems to have been a surge of articles from 1999 [18]. In 2010, the International Medical Informatics Association (IMIA) revised the earlier (2000) international recommendations for health informatics or medical informatics education with the hope that these would help to establish courses and perhaps lead to sharing of courseware [18]. The revised 2010 recommendations were designed to meet the educational needs of health professionals from medicine, nursing, health care management, dentistry, pharmacy, public health, health record administration, and informatics/computer science and for dedicated programs in biomedical and health informatics [18]. This indicates a broad health professional reach. Despite the direction provided in the IMIA recommendations, there is still evidence that embedding informatics within a health professional education program is not commonplace [19].

A survey of medical schools in the United Kingdom identified that 17% of the 76% of medical schools that responded had little or no health informatics included in their curricula, and this is despite the General Medical Council’s curriculum requirements [20]. Similarly, in pharmacy schools in the United States, it was noted that little “progress had been made in pharmacy school curricula in response to the increasing importance of informatics to the profession” [21]. A global approach was suggested to provide flexible, Web-based, and standards-based medical informatics education, but this does not seem to have been well accepted [22]. A common issue has been the lack of suitably prepared faculty to teach health informatics [18,20-22].

For more than 30 years, nursing has had an interest in nursing informatics competencies [23]. However, early publications often described the use of computers by nurses and focused on computer skills and what should be included in nursing education [24]. An international initiative, driven from the United States, the Technology Informatics Guiding Education Reform (TIGER) developed competencies to guide the nursing profession, but these are not widely used [25]. Many countries have considered nursing informatics competencies for their nurses [26-29], recognizing that their context and needs may be particular to their country. More recently, the IMIA Nursing Informatics Special Interest Group focused on nursing informatics competencies at a postconference meeting of world leaders [30]. The aim of this meeting was to “publish a set of informatics competency recommendations for nurses educated in the next decade that cover the informatics skills required for improved, innovative and even transformative health and health care delivery” [23]. A comprehensive scope was set by including information management and the use ICT for aspects such as electronic health records, medical devices, telemedicine, patient portals, electronic health, and mobile apps, with the hope that the competencies would also help prepare nurses for future developments.

Despite the use of ICT in health care practice becoming increasingly commonplace in developed countries, nursing education has lagged in providing the preparation needed for new nurses to be aware and have the opportunity to develop the knowledge, skills, and attitudes they will need in practice. As Murphy and Goosen state, “After almost 25 years it is still problematic how few schools of nursing offer education on how the values of patient focused care can be mixed with careful application of health informatics tools and good professional information management” [23].

### New Zealand as a Context

New Zealand provides a context for this project, and this section includes some of the New Zealand health informatics history that helped to shape the current informatics landscape. Despite early recognition of the need for nursing informatics competencies and guidelines to inform practice and undergraduate nurse education in New Zealand, the embedding of nursing informatics within the nursing curricula did not occur [13,31].

In 1989, a nurse educator, Jan Hausman, was seconded by the Ministry of Education to develop New Zealand guidelines for teaching nursing informatics [14]. However, this early initiative saw little change in the nursing curricula. Nevertheless, a growing interest in nursing informatics started around this time, and by 1991, a national nursing informatics group (Nursing Informatics New Zealand [NINZ]) was formed [31]. In the mid-1990s, this group developed and published Standards for Nursing Informatics with the notion that these guidelines would guide nursing practice [13]. Unfortunately, these guidelines were not widely adopted.

In 2000, NINZ joined the New Zealand Health Informatics Foundation to form Health Informatics New Zealand (HiNZ), a not-for-profit organization that supports the field of health informatics, with a focus on events and professional development in New Zealand. HiNZ members include health professionals (including nurses), health sector managers, ICT experts, industry managers, academics, students, and government personnel [32].

In 2006, a report identified that more people, particularly those already in the health workforce, needed to be trained in health informatics [33]. Subsequently in 2012, based on this work and that of IMIA [18], a cross-institutional group of New Zealand informatics educators collaborated to develop “Core Competencies for Health Informatics” under the umbrella of HiNZ [34,35]. These competencies were designed for the existing workforce and were directed to health professionals, managers, and technical experts in health care. An outcome was government recognition and funding of *primer* workshops based around introductory health informatics concepts that were delivered around the country to local health care organizations. It was hoped that in providing the *primer* workshops, more health care workers would be informed and engaged in health informatics, and this would, in the longer term, address the shortage of health professional champions and interdisciplinary team members trained in health informatics [34,35].

However, these efforts and the core competencies, although they included health professionals, were focused toward those already in practice and not those in training. A project to address this gap, specifically for nurses, commenced in 2016.

## Methods

To identify the nursing competencies needed for New Zealand nurses, a project was initiated in 2016 by a team of 3 nurse educators from 3 different schools of nursing. The objective of this project was to use a case study approach to develop nursing informatics guidelines specific for the New Zealand context that were based on principles encompassing key knowledge, skills, and behaviors for student nurses to attain over the time of their undergraduate education to be ready to begin practice as a registered nurse (RN). A case study approach was selected as it allowed for descriptive and exploratory analysis [36]. In addition, any nursing informatics guidelines needed to align with the New Zealand Nursing Council competencies for RNs [37]. Over the next 2 years, evidence-based Guidelines for Nursing Informatics Competencies for Undergraduate Nurses in New Zealand were developed, and this work is now published and ready for dissemination [38]. Preceding this, in 2015, a study mapping the TIGER competencies [25] against the current legislation and practice in New Zealand was conducted, but the feeling was that the US-centric competencies did not suit the New Zealand health care and educational context [15,39]. This work was presented at the national nursing informatics conference in New Zealand, which identified other interested nurses and resulted in a collaboration to consider what nursing informatics competencies were needed for New Zealand nurses. The first stage of the collaboration was between nurse lecturers from 3 different schools of nursing and consisted of mapping their respective school’s existing undergraduate nursing curricula against the Australian nursing informatics standards [26]. This work identified gaps in the existing undergraduate curricula for each school [12,40]. To address this gap and identify the nursing competencies needed for New Zealand nurses, a review of literature and then iterative consultation with an advisory group and key stakeholders was undertaken. The project team met regularly (usually virtually) and collaboratively created the foundation for the guidelines for nursing informatics competencies for undergraduate nurses in New Zealand, which were shaped by feedback received from the advisory group and key stakeholders until the final product was developed: *Guidelines: Informatics for nurses entering practic*e [38].

## Results

The *Guidelines: Informatics for nurses entering practice* (hereafter called The Guidelines) identify 5 health informatics principles for nurses at the end of their undergraduate nursing education program as they enter practice as level 1 or novice RNs [38] (Textbox 1).

**Figure 1:**
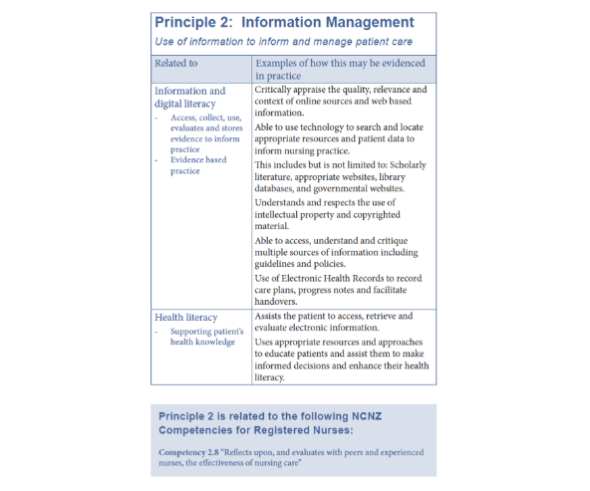
The Guidelines showing principle 2. NCNZ: Nursing Council of New Zealand.

The Guidelines identify the key knowledge, skills, and behaviors toward nursing informatics for nurses as they enter practice as an RN, and as such, they have been developed and articulated to inform undergraduate nursing education. The principles are explicitly aligned to the Nursing Council of New Zealand (NCNZ) Competencies for RNs [37] (Figure 1).

Background literature that informed The Guidelines included reports from international nursing informatics initiatives including from Australia (the Australian National Informatics Standards for Nurses and Midwives) [26], from the initiative driven from the United States (TIGER) [25], from the Royal College of Nursing in England (Every nurse an e-nurse: Digital capabilities for 21st century nursing) [29], and from Canada (Nursing informatics entry to practice competencies for RNs) [27].

This project was informed by an advisory group of 12 nurse leaders from practice, education, policy, the nursing regulatory body, and industry. Drafts of The Guidelines were distributed, and feedback was sought using an iterative process. In addition, 4 nursing organizations were asked to be kept informed: The Office of the Chief Nurse in the Ministry of Health at the government level; the regulatory body, NCNZ; and The Council of Deans and Nurse Educators in the Tertiary Sector, from the education sector.

In addition, the formatting was considered, and alongside each principle, examples were provided. The inclusion of examples from everyday practice in the New Zealand health system adds a local context (Figure 1). Finally, a glossary was added so that terms are defined, providing a common understanding.

**The 4 principles.**

**Principle 1: Professional practice**

Nurses are accountable and responsible for their use of information and communication technologies (ICT)

**Principle 2: Information management**

Use of information to inform and manage patient care

**Principle 3: ICT to enhance the health of New Zealanders**

Nurses effectively use ICT to assist with the delivery of quality nursing care to improve patient outcomes

**Principle 4: General computer and ICT skills**

The nurse is adaptable in different health care environments through transferrable ICT skills

## Discussion

### Principal Findings

This project aims to inform, influence, and potentially change professional nursing practice and policy through providing clear guidelines for nursing informatics competencies. The Guidelines align with the NCNZ competencies for RNs [37], which all schools of nursing in New Zealand work with the development of their curricula. Furthermore, this project supports the national health strategy as nurses provide health care service to support people in New Zealand to “Live well, Stay well, Get well,” which is a key component of the 2016 New Zealand Health Strategy [3].

By considering the past and previous attempts to introduce nursing informatics competencies in New Zealand, there is an opportunity to learn and to improve on earlier endeavors. A common problem faced by earlier efforts includes moving from the creation of guidelines to a position where competencies are recognized and inform policy to where they are embedded in education. This project supports actively building connections between the tertiary education providers within the schools of nursing, thereby crossing perceived divisions between universities, polytechnics, and institutes of technology. By demonstrating engagement within the nursing communities, specifically nurse lecturers and the key nursing regulatory and policy stakeholders, The Guidelines may be acceptable to all and are more likely to be accepted and influence policy.

In terms of policy impact, this project has the potential to influence the NCNZ to explicitly address the inclusion of nursing informatics within undergraduate nursing curricula. The implementation of these guidelines nationally would impact all 17 schools of nursing in New Zealand by ensuring that present and future RNs are consistently prepared for working in a technological age. This will likely have flow-on effects for patient care, potentially improving safety and efficiency within the health care system and improving quality of care for recipients, while acknowledging the role of nurses in health maintenance and health promotion as well as providing health care for those who are sick or dying. Technology is used in all aspects of health care, and ensuring that our workforce is adequately prepared for its use in health care has the potential to assist all recipients of care, including Māori—the indigenous people of New Zealand—and those from neighboring Pacific Islands, who are overrepresented in New Zealand’s worst health statistics [3].

The impact of policy in terms of education will affect nursing students, who remain predominantly women (9% of the current nursing workforce is male) [1]. Understanding the use of technology in health care will better prepare them to be competent and effective future nurses. The Guidelines form a bridge between the theory of nursing informatics, education of nurses, and clinical practice.

### Next Steps

The challenge is to now disseminate this work to the New Zealand nursing education community so that The Guidelines can be incorporated into nursing curricula. The first step is to share The Guidelines across all schools of nursing in New Zealand so that nurse educators can consider how The Guidelines can be implemented into their undergraduate programs of nursing, which will facilitate effective knowledge transfer and uptake of The Guidelines into practice. Furthermore, there is a need to identify and then address the concerns, barriers, and facilitators to using The Guidelines in nursing education. In addition, from a New Zealand perspective, where the indigenous people are Māori, there is a need to understand any issues specifically from the perspectives of Māori as concerns related to data guardianship and nurses acting as kaitiaki/guardians of the data may apply [41].

### Conclusions

Despite local and international efforts to include health informatics as part of the curricula for preparing health professionals for practice in the 21st century, there remains inconsistency in achieving this. This case study illustrates the development of heath informatics competencies and guidelines for one profession, in one country. The challenge is moving from educational rhetoric to practice and policy to ensure that health informatics is embedded into the educational preparation for all health professionals. The importance of learning from past initiatives is highlighted. The process used to develop the “Guidelines: Informatics for nurses entering practice” for use in New Zealand is expected to guide undergraduate nursing education and as such form a bridge between theory, education, and practice.

## Abbreviations

HiNZ: Health Informatics New Zealand
ICT: information and communication technologies
IMIA: International Medical Informatics Association
NCNZ: Nursing Council of New Zealand
NINZ: Nursing Informatics New Zealand
RN: registered nurse
TIGER: Technology Informatics Guiding Education Reform
